# Supplementary material for: Novel Driver Strength Index highlights important cancer genes in TCGA PanCanAtlas patients
Source: PeerJ. 2022 Aug 11;10:e13860. doi: 10.7717/peerj.13860 (PMC9375969; doi:10.7717/peerj.13860)
Supplement: Supplemental Information 2 [file peerj-10-13860-s002.zip › COHORTS/genes plots/detailed/2021_8_16_14_9_distribution_events_detailed_males_14.pdf]

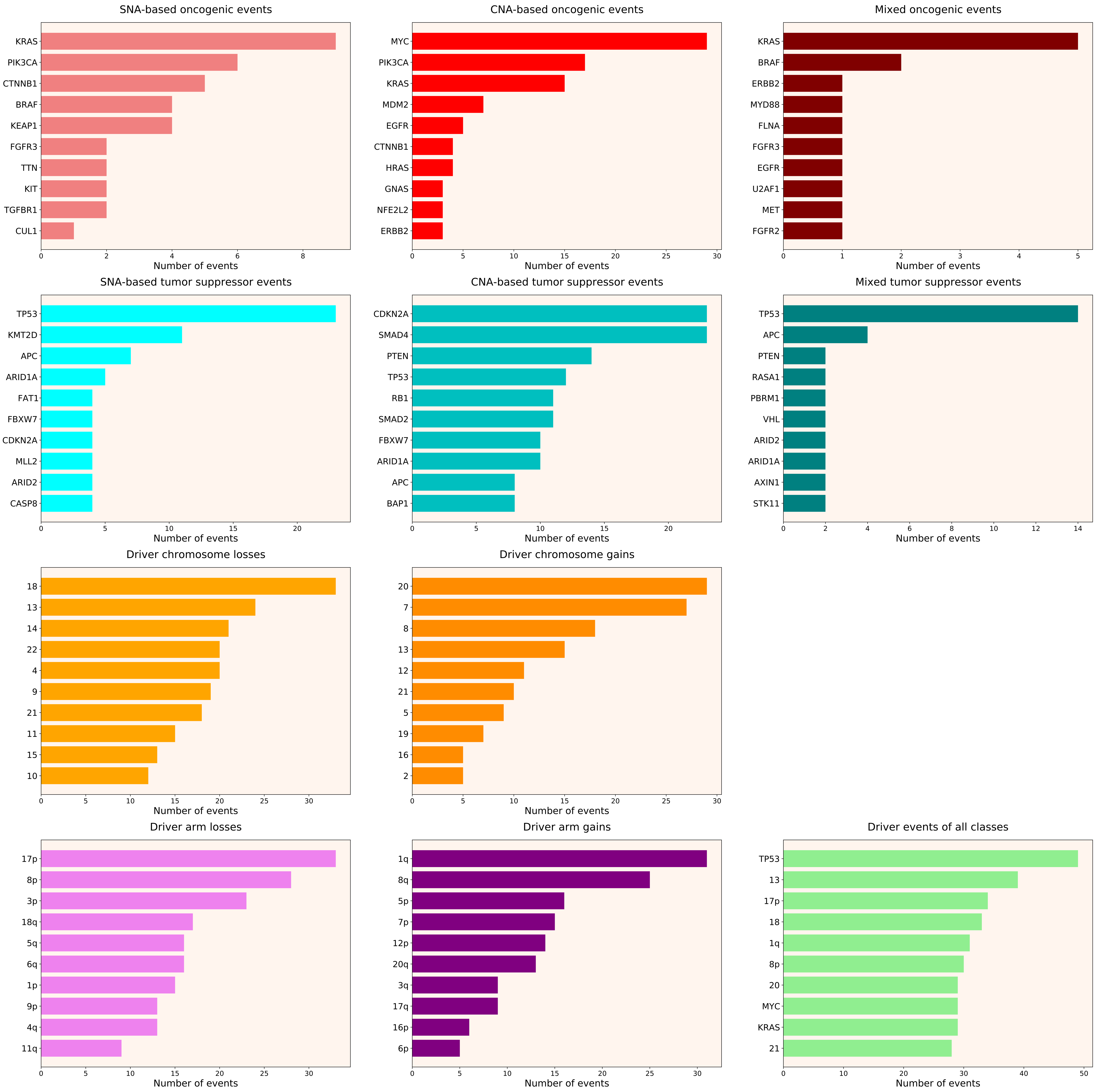
SNA-based oncogenic events
KRAS
PIK3CA
CTNNB1
BRAF
KEAP1
FGFR3
TTN
KIT
TGFBR1
CUL1
Number of events
CNA-based oncogenic events
MYC
PIK3CA
KRAS
MDM2
EGFR
CTNNB1
HRAS
GNAS
NFE2L2
ERBB2
Number of events
Mixed oncogenic events
KRAS
BRAF
ERBB2
MYD88
FLNA
FGFR3
EGFR
U2AF1
MET
FGFR2
Number of events
SNA-based tumor suppressor events
TP53
KMT2D
APC
ARID1A
FAT1
FBXW7
CDKN2A
MLL2
ARID2
CASP8
Number of events
CNA-based tumor suppressor events
CDKN2A
SMAD4
PTEN
TP53
RB1
SMAD2
FBXW7
ARID1A
APC
BAP1
Number of events
Mixed tumor suppressor events
TP53
APC
PTEN
RASA1
PBRM1
VHL
ARID2
ARID1A
AXIN1
STK11
Number of events
Driver chromosome losses
18
13
14
22
4
9
21
11
15
10
Number of events
Driver chromosome gains
20
7
8
13
12
21
5
19
16
2
Number of events
Driver arm losses
17p
8p
3p
18q
5q
6q
1p
9p
4q
11q
Number of events
Driver arm gains
1q
8q
5p
7p
12p
20q
3q
17q
16p
6p
Number of events
Driver events of all classes
TP53
13
17p
18
1q
8p
20
MYC
KRAS
21
Number of events
